# Use of unsupervised machine learning to characterise HIV predictors in sub-Saharan Africa

**DOI:** 10.1186/s12879-023-08467-7

**Published:** 2023-07-19

**Authors:** Charles K. Mutai, Patrick E. McSharry, Innocent Ngaruye, Edouard Musabanganji

**Affiliations:** 1 African Center of Excellence in Data Science, University of Rwanda, Kigali, BP 4285 Rwanda; 2 Department of Mathematics, Physics and Computing, Moi University, Eldoret, Kenya; 3 College of Engineering, Carnegie Mellon University Africa, Kigali, BP 6150 Rwanda; 4 Oxford-Man Institute of Quantitative Finance, Oxford University, Oxford, OX2 6ED UK; 5 College of Science and Technology, University of Rwanda, Kigali, Rwanda; 6 College of Business and Economics, University of Rwanda, Kigali, Rwanda

## Abstract

**Introduction:**

Significant regional variations in the HIV epidemic hurt effective common interventions in sub-Saharan Africa. It is crucial to analyze HIV positivity distributions within clusters and assess the homogeneity of countries. We aim at identifying clusters of countries based on socio-behavioural predictors of HIV for screening.

**Method:**

We used an agglomerative hierarchical, unsupervised machine learning, approach for clustering to analyse data for 146,733 male and 155,622 female respondents from 13 sub-Saharan African countries with 20 and 26 features, respectively, using Population-based HIV Impact Assessment (PHIA) data from the survey years 2015–2019. We employed agglomerative hierarchical clustering and optimal silhouette index criterion to identify clusters of countries based on the similarity of socio-behavioural characteristics. We analyse the distribution of HIV positivity with socio-behavioural predictors of HIV within each cluster.

**Results:**

Two principal components were obtained, with the first describing 62.3% and 70.1% and the second explaining 18.3% and 20.6% variance of the total socio-behavioural variation in females and males, respectively. Two clusters per sex were identified, and the most predictor features in both sexes were: relationship with family head, enrolled in school, circumcision status for males, delayed pregnancy, work for payment in last 12 months, Urban area indicator, known HIV status and delayed pregnancy. The HIV positivity distribution with these variables was significant within each cluster.

**Conclusions /findings:**

The findings provide a potential use of unsupervised machine learning approaches for substantially identifying clustered countries based on the underlying socio-behavioural characteristics.

**Supplementary Information:**

The online version contains supplementary material available at 10.1186/s12879-023-08467-7.

## Introduction

One of the most threatening infectious diseases and a burden on public health globally is HIV. Global estimates for 2019 show that 38 million people are living with HIV, while 1.7 million and 690,000 new infections and deaths are reported, respectively. This is despite the fact that diagnosis and access to antiretroviral therapy have made great strides in recent years (ART). East and Southern Africa account for more than half of all HIV-positive individuals, 42.9% of new infections, and 43.5% of AIDS-related fatalities [1]. Various estimates of the number of people living with HIV in 2021 ranged from 220,000 to 1.7 million in Namibia and Tanzania, respectively; 4,300 and 54,000 new HIV infections in Rwanda and Tanzania, respectively; and 4,300 and 54,000 deaths from AIDS-related illnesses in Rwanda and Tanzania, respectively [2]. By 2030, the Joint United Nations Program (UNAIDS) aimed to eradicate AIDS as a global health threat [3, 4]. The COVID-19 pandemic, nevertheless, has already reversed the gains made, and it may even have had a negative effect by raising the death toll from AIDS in sub-Saharan Africa [5].

Despite considerable HIV prevention programs in East and Southern Africa, the HIV epidemic widely spread throughout the region [6, 7]. Significant variations exist between SSA countries in HIV incidence and prevalence and complicate the creation of effective interventions, which include extensive intra- and inter-national socio-behavioural and cultural diversity. Certain populations require targeted responses to address and help safeguard them based on the granular facts about the HIV epidemic [8]. Contrary to a homogeneous distribution of resources, strategies are designed to maximize resource allocation and, consequently, have a higher impact and level of efficiency in identifying the people who are most susceptible to infection [9, 10].

Social-behavioural characteristics are among the most significant predictors of HIV transmission, so it is crucial to study how they affect the HIV epidemic in a specific community [11]. Including social-behavioural HIV predictive indicators in the analysis may significantly improve the recognition of and maybe cluster countries at higher risk of infection, boosting the best screening options and assisting with HIV testing and counselling.

By taking into account the type of variable, the scale of measurements, and the subject matter knowledge, cluster analysis is a technique for grouping variables based on their similarity or distance. Objects in one group are meant to be similar, while those in other groups are meant to be somewhat distinct [12].

The clustering of HIV infections in Kenya has been studied in the past using the Kulldorff-scan approach [13]. Moreover, Tanser et al. used the same method to locate infection clusters [14]. Ying et al. examined biological and behavioural connections using the Kulldorff technique to detect geographic clusters of HIV in Ethiopia [15]. Additional studies that used Kulldorf methods to conduct geographic clustering at the national level include the Kulldorff-scan and Moran’s Index approach to assess the spatial distribution of newly diagnosed HIV-positives in Kenya, Ethiopia and, respectively [16]. Other studies that have also used Kulldorf include, Oliveira et al. [17] in which they mapped geographical areas confirming the existence of heterogeneity. Cuadros et al. similarly used the Kulldorff spatial scan test to identify and map the geographic distribution of HIV infection throughout sub-Saharan Africa (SSA) and highlighted priority geographic regions for HIV programs.

However, the approach did not indicate social behavioural variable characteristics but they hypothesised clustering was a reflection of differences in particular behavioural and biological variables amongst sub-populations, amplifying more pronounced inequalities in HIV prevalence [18].

In the Amhara region of Ethiopia, Gelaw et al. employed a Bayesian conditional autoregressive model to perform geographical clustering and linkage between HIV infection and socio-demographic factors [19]. According to their research, immigrants and those with poor levels of education were linked to greater HIV cluster risk. Biressaw et al. used principal component analysis to cluster HIV patients into three clusters and found a 78% variation in their data [20].

These techniques, however, do not inform on how regional variations in HIV risk factors differ or which specific socio-behavioural patterns at the regional level are connected to different county-level rates of new HIV infections. The requirement for household weights limits the application of Kulldorff-scan methods, and the strategy ignored social behavioural traits even though it was expected that clustering was a reflection of variances in some behavioural and biological elements.

Recent studies have demonstrated the use of unsupervised machine learning in clustering analysis. Hierarchical clustering, which is an unsupervised, machine learning has been used by Andresen et al. to identify subgroups of males who engage in sexual activity with other men who exhibit similar sexual behaviour, and taking these groups into account in addition to traditional risk variables improved predictions of who will be diagnosed with Sexually Transmitted Infections (STI) [21]. It was also utilized by Xu et al. to discover subject clusters with word groupings, frequencies, and attributes relevant to user chats associated with HIV [22]. Unsupervised machine learning was employed by Farooq et al. to summarize and cluster HIV viral load patterns [23]. Jonathan et al. applied unsupervised learning approaches as well to identify pregnancy co-morbidities [24]. Merzouki et al. identified populations in Malawi that share a common risk of getting HIV using latent class analysis [25].

Merzouki et al. discovered that socio-behavioural parameters play a significant role in predicting the trajectory of the HIV epidemic while utilizing DHS data to group SSA countries based on socio-behavioural characteristics [26].

The purpose of this study is to use unsupervised machine learning techniques to identify the homogeneity of countries based on socio-behavioural predictors of HIV for screening that was identified in the previous study [27]. Policymakers can get important insights for developing targeted policies and interventions by identifying groups of counties with similar socio-behavioural traits. Sharing experiences, best practices, and lessons gained among counties within a cluster promotes mutual learning and improves decision-making. Using clusters permits comparison study between counties, highlighting similarities and differences in socio-behavioural qualities and providing illuminating data on the influences of sociological, cultural, and economic factors on various dimensions of growth and well-being. With the ability to prioritize according to each cluster's unique needs, effective resource allocation will have an impact. Patterns found inside clusters provided insight into future behaviour and development paths, allowing for the planning of prospective obstacles.

## Methods

### Data

This study made use of data from the Population-based HIV Impact Assessment (PHIA) project, which comprises cross-sectional household-based surveys made to evaluate HIV-related important health indicators [28]. PHIA conducted surveys in 13 countries: Côte d’Ivoire (2017–2018), Cameroon (2017–2018), Ethiopia (2016–2017), Eswatini (2016–2017), Kenya (2018), Lesotho (2016–2017), Zimbabwe (2015–2016), Malawi (2015– 2016), Namibia (2017), Rwanda (2018–2019), Tanzania (2016–2017), Uganda (2016–2017) and Zambia (2016). The PHIA survey has been covered in more depth elsewhere [23].

In this study, the data from 13 PHIA country surveys were merged adult datasets with HIV test results to obtain two sets of data, comprising 146,733 male and 155,622 female respondents, respectively (Table 1).

**Table 1:** HIV prevalence and the number of individuals included in PHIA survey

	**HIV prevalence**		**Number of individuals included in PHIA survey**	
**Country**	**Male**	**Female**	**Female**	**Male**
All countries	6.4	10.2	155,622	146,733
Tanzania	3.5	6.1	17,476	16,584
Rwanda	2.1	3.6	16,015	14,700
Uganda	4.8	7.9	15,822	14,131
Cameroon	2.5	4.9	14,178	13,434
Zimbabwe	11.6	15.2	13,240	11,794
Zambia	9.6	14.4	10,994	10,286
Ethiopia	2	4	10,058	10,112
Malawi	8.8	12.3	10,242	9,587
Cote d'Ivoire	1.7	3.7	9,274	9,653
Namibia	9.8	15.7	9,705	9,091
Lesotho	20.4	30.7	6,488	6,584
Swaziland	21	32.5	6,393	5,482
Kenya	3.1	6.5	15,737	15,295

The variables that were identified in the previous study [27] as the HIV predictors for screening in SSA were included in our study. These include both quantitative predictors (age, age at first sex, wealth score, number of pregnancies for women, number of pregnancies for men) and qualitative predictors (ever sought Tuberculosis (TB) treatment, relationship with the head of the family, ever enrolled in school, the highest level of education, work for pay in the last 12 months, delaying or avoiding pregnancy, ever sought TB treatment, urban area indicator, marital status and status of circumcision for men, known HIV status for women). Mutai et al. [27], provide further information on the procedures used to process the data.

Each dimension matched a certain attribute expressed as a percentage or average, and there were 20 and 26 dimensions for males and females, respectively, to represent each country (Table 2, Table S1, and Table S2).

**Table 2:** Socio-behavioural predictors of HIV that are included in the analysis

Variable	Categories	Males	Females
Average age of respondent		31.8	32.1
work for payment last 12 months (%)	no	45.5	66.3
	yes	54.5	33.7
Ever married or lived together (%)	no	43.5	30.6
	yes	56.5	69.4
Delaying or avoiding getting pregnant (%)	no	55.2	57
	yes	44.6	42.5
Circumcision status (%)	yes	60.3	
	no	39.7	
First age engaging at sex		18.2	17.9
Ever visited TB clinic for treatment (%)	no	92.2	92.5
	yes	7.7	7.3
Urban area indicator (%)	rural	55.7	56
	urban	44.3	44
Average wealthscorecont		0.4	0.5
Relationship to household head (%)	Brother/sister	4.6	3.9
	Grandchild	4.1	3.2
	Head	51.8	26
	Not related	3.2	2.5
	other relative	6.1	5.9
	son or daughter	23.7	2.7
	wife/husband/partner	4.3	35.2
	parent		1.3
Enrolled in school (%)	no		76.9
	yes		17.6
Average number of times been pregnant			3.1
Average number of children had since 2012			0.7

### Analysis

We used Principal Component Analysis (PCA), which is a dimensionality reduction approach often employed in data analysis and machine learning. It reduces a dataset with numerous variables to a more manageable collection of uncorrelated variables known as principal components (PC) [23, 24]. It was utilised to reduce the dimensionality of a dataset related to socio-behavioural factors in sub-Saharan African (SSA) countries. The original dataset had 20 and 26 variables (dimensions), respectively, for each sex, and we applied PCA separately for each sex to reduce the dimensions to two.

Reducing dimensionality enables comparison and display of patterns or similarities among SSA countries based on socio-behavioural HIV indicators. The two PCA-derived dimensions minimize information loss while retaining the most variance from the original dataset. As a result, we plotted the data on a two-dimensional scatter plot, where each point corresponds to a distinct country and is positioned based on the values of the two principal components.

The simplified representation of the original data using the reduced dataset with two dimensions per sex preserves as much information as possible. The subsequent analysis or interpretation of the data was made easier by this visualization, which helped in identifying groupings or similarities in the socio-behavioural characteristics among SSA countries [29].

To agglomerative hierarchical clustering, a pairwise country dissimilarity measure was calculated using the Euclidean distance (a metric for determining the straight-line distance between two points in multidimensional space). Here, data points in a multidimensional space were used to represent the socio-behavioural characteristics of each country. Using the values of each country's socio-behavioural attribute, the Euclidean distance between each pair of countries was then determined. Given that each country is represented by an n-dimensional vector, the dissimilarity (d_i,j_) between two countries i, j is measured using the Euclidian distance, which is as follows:

$${d}_{i,j}=\sqrt{{\sum }_{k=1}^{n}{\left({C}_{i,k}-{C}_{j,k}\right)}^{2}}$$

where *n* is the total number of variables used in our analysis to describe a country. The C_i,k_ and C_j,k_ are the k^th^ elements of *n*-dimensional vectors C_i_ and C_j_, respectively.

A dendrogram, a tree-like diagram that depicts the clustering procedure and demonstrates the hierarchical links between the groups, was then used to exhibit the results of the hierarchical clustering. By observing the degrees of dissimilarity at which clusters merge, the dendrogram can assist in determining the ideal number of clusters.

We assessed the degree of dissimilarity between clusters using Silhouette Index; a metric for evaluating the efficiency of clustering results, considering both the compactness and the separation between clusters, ranges from -1 to 1, where a value near 1 denotes effective clustering, a value We assessed the degree of dissimilarity between clusters using Silhouette Index; a metric for evaluating the efficiency of clustering results, considering both the compactness and the separation between clusters, ranges from -1 to 1, where a value near 1 denotes effective clustering, a value near 0 denotes overlapping or poorly separated clusters, and a value near -1 denotes ineffective clustering. Therefore, the number of clusters with the highest optimal configuration index was selected.

For each observation (i.e. country) c_i_ the silhouette width sil(c_i_) is defined as;

$$sil\left({c}_{i}\right)=\frac{b\left({c}_{i}\right)-a\left({c}_{i}\right)}{\mathrm{max}(a\left({c}_{i}\right),b\left({c}_{i}\right))}$$

where a(c_i_) is the mean dissimilarity between c_i_ and all other points (i.e. countries) of the cluster to which c_i_ belongs, and

$$b\left({c}_{i}\right)=d\left({c}_{i,} {C}_{closest}\right)=\mathrm{min}(d\left({c}_{i,}C\right))$$

is the dissimilarity between c_i_ and its closest cluster C_closest_, with d(c_i,_ C) being the mean distance from c_i_ to all observations of cluster C to which it does not belong. The silhouette index is then obtained by averaging the silhouette widths over the whole data set:

$$SI={\sum }_{i=1}^{m}sil\left(ci\right)$$

where *m* is the total number of countries included in the analysis.

Box plots then, were used to visualise the distribution (median) of HIV prevalence within each cluster and compared with the various countries within the identified clusters.

## Results

We analysed data from 146,733 men and 155,622 women, ranging from 6,393 women and 5,482 men in Swaziland to 17,476 women and 16,584 men in Tanzania. Males had an HIV prevalence of 6.4%, while females had an HIV prevalence of 10.2%. Men's HIV prevalence ranged from 1.7% in Cote d'Ivoire to 21.0% in Swaziland, and women's prevalence ranged from 3.6% in Rwanda to 32.5% in Swaziland. These differences were seen throughout all the countries (Table 1). Socio-behavioural indicators also varied significantly between the 13 countries (Table 2, Table S1, and S2).

**Fig. 1:**
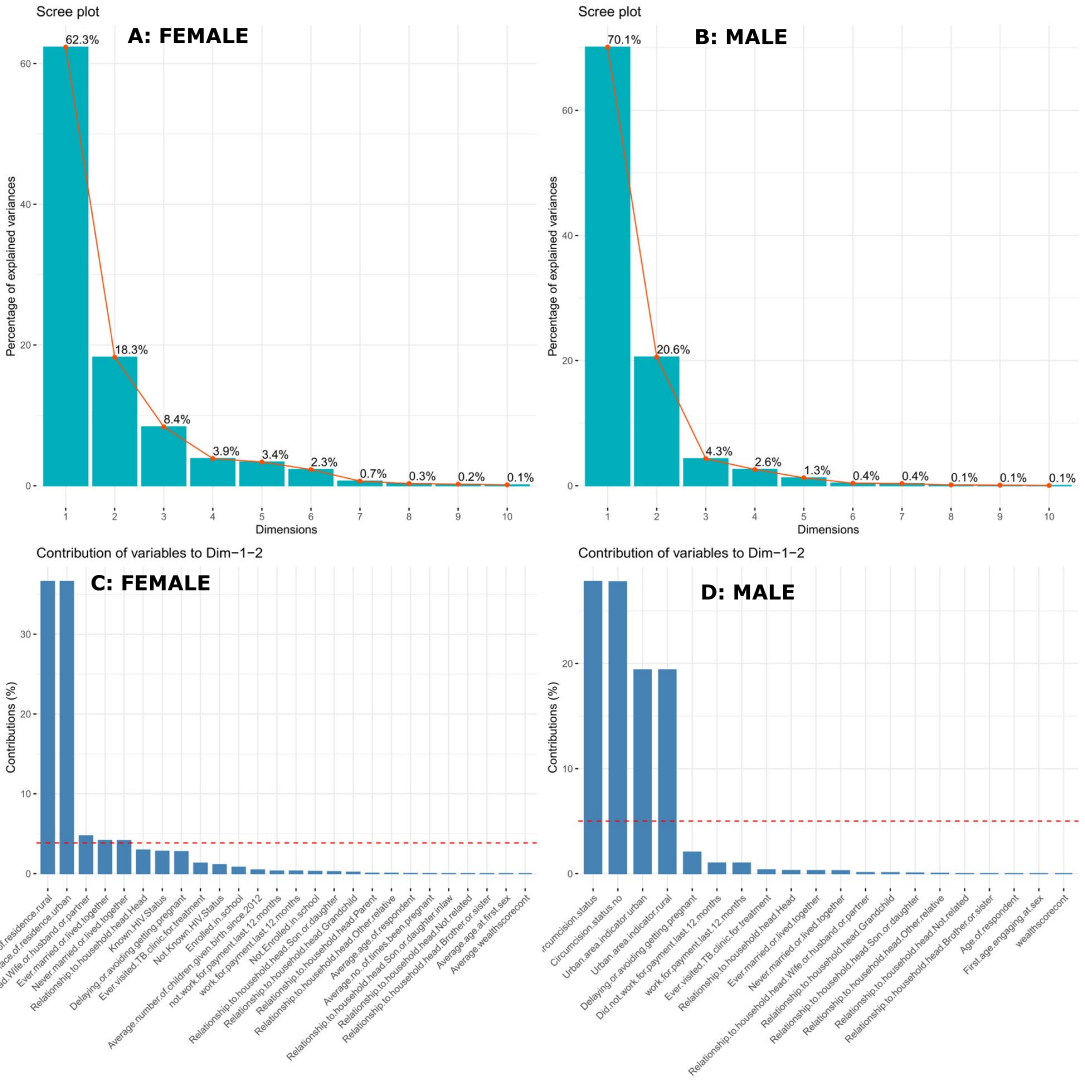
Scree plots (**A**: Female and **B**: Male) and variables contribution to principal components 1 to 2 (**C**: Female, **D**: Male)

**Fig. 2:**
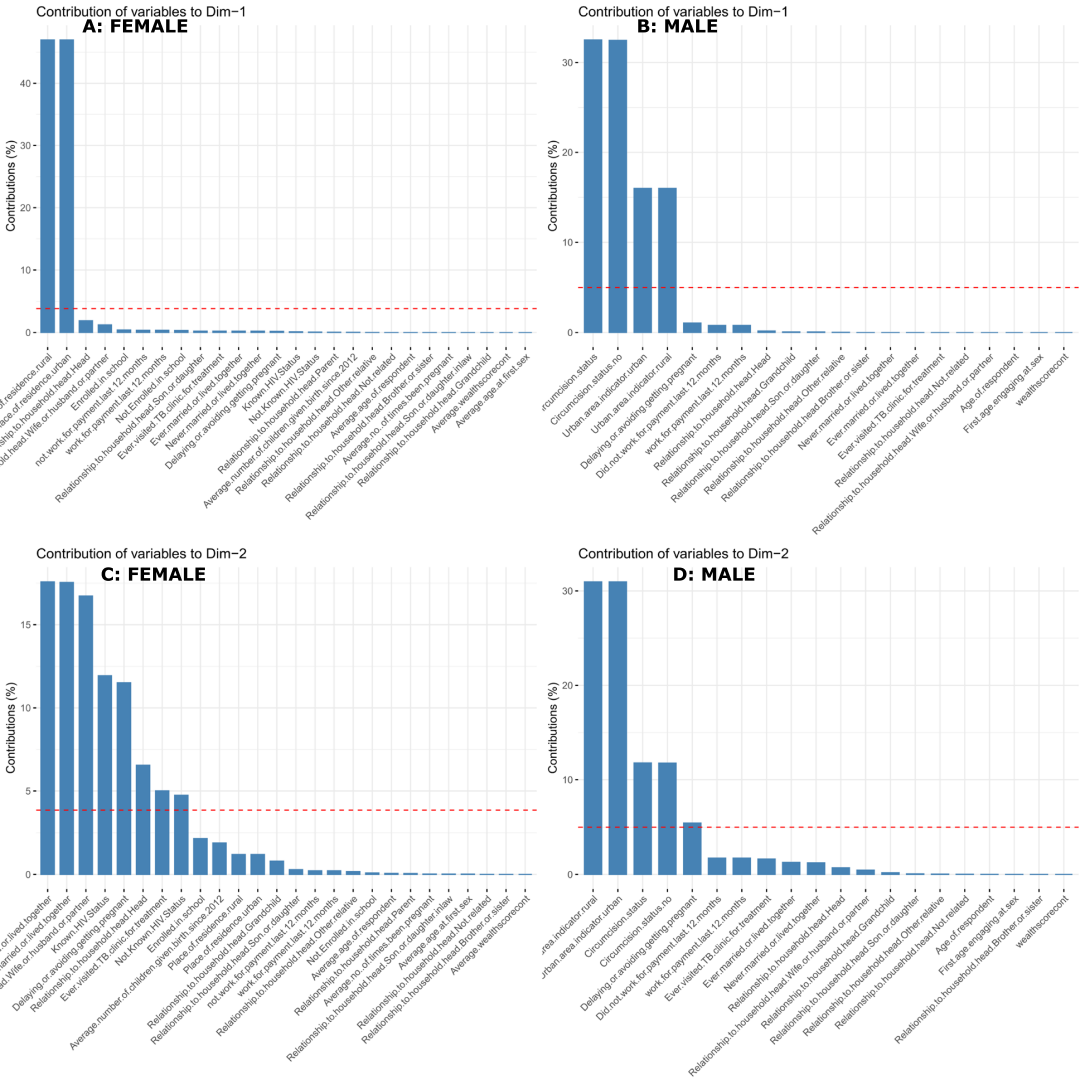
Variables contribution to principal components 1 (**A**: Female, **B**: Male,) and 2 (**C**: Female, **D**: Male)

Using PCA, we found that the first principal component described 62.3% and 70.1% of the total socio-behavioural variation across 13 countries, and the second principal component explained 18.3% and 20.6% of the variance among the 26 and 20 variables examined in females and males, respectively, (Fig. 1, Fig. 2 and Tables S3, S4). Male circumcision status (31.5% for both circumcised and uncircumcised males) and place of residence (16% for both urban and rural areas) were the original socio-behavioural variables that contributed most to the first principal component (Fig. 2, B). Delaying or preventing conception contributed 5.5%, both urban and rural dwellers contributed 31%, and both circumcised and uncircumcised males contributed 12% to principal component two, (Fig. 2, D).

The original socio-behavioural factors in females that made the biggest contributions to the first principal component were a place of residence (47% for both rural and urban) (Fig. 2, A), while the second principal component contributions were ever married (17.5% for both married or living together and not married or living together), known HIV status (12% for known status and 4% for not known status), 11.5% for females delaying or avoiding pregnancies, and 5% for those who had ever visited a TB clinic and relations to household head (16.5% for being wife or partner and 6.5% for head), (Fig. 2, C).

**Fig. 3:**
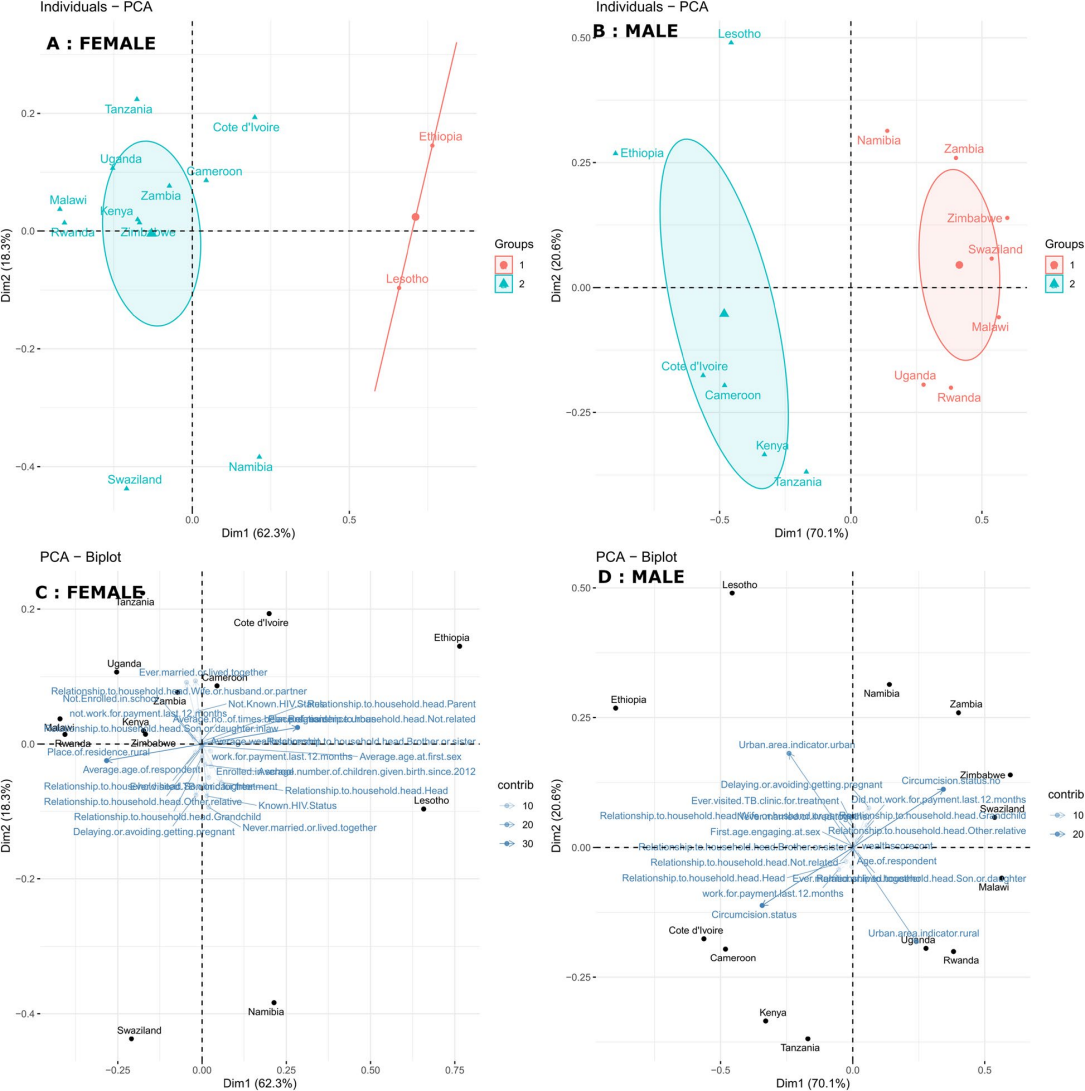
Two-dimensional scatter plot projection of countries and their corresponding variables' contribution to PCA

Projecting the 13 SSA countries in two dimensions (Fig. 3). These show how the original socio-behavioural variables vary over the two-dimensional space. At the top left branch (Fig. 3, A) are groups 2 of countries, such as Cameroon, Côte d'Ivoire, Kenya, Malawi, Namibia, Zambia, Rwanda, Swaziland, Tanzania, Uganda and Zimbabwe, lying next to each other. Inthese counties (Fig. 3, C), more women are married or cohabitating, yet a significant proportion are uneducated women and are unemployed in the last 12 months. The majority of them are related to the household's head as a wife or partner, live in rural areas and are not aware of their HIV status.

On the right quadrant (Fig. 3, A) are females in group 1 countries, Ethiopia and Lesotho, where there is a higher enrollment in school, ever visited TB clinic for treatment, the majority know their HIV status, never married or lived together, they are head of the family, most had jobs in the last 12 months and live in urban dwellings (Fig. 3, C).

For their male counterparts, (Fig. 3, B), Namibia, Malawi, Rwanda, Uganda, Swaziland,

Males living in countries like Cameroon, Côte d'Ivoire, Ethiopia, Kenya, Lesotho and Tanzania are represented as group 2 on the left branch (Fig. 3, B). These countries share traits (Fig. 3, D) like a high rate of male circumcision, a higher percentage of them are living in urban areas, were employed in the last 12 months and the majority of them are household heads.

### Clustering and distribution of HIV prevalence among the clusters

**Fig. 4:**
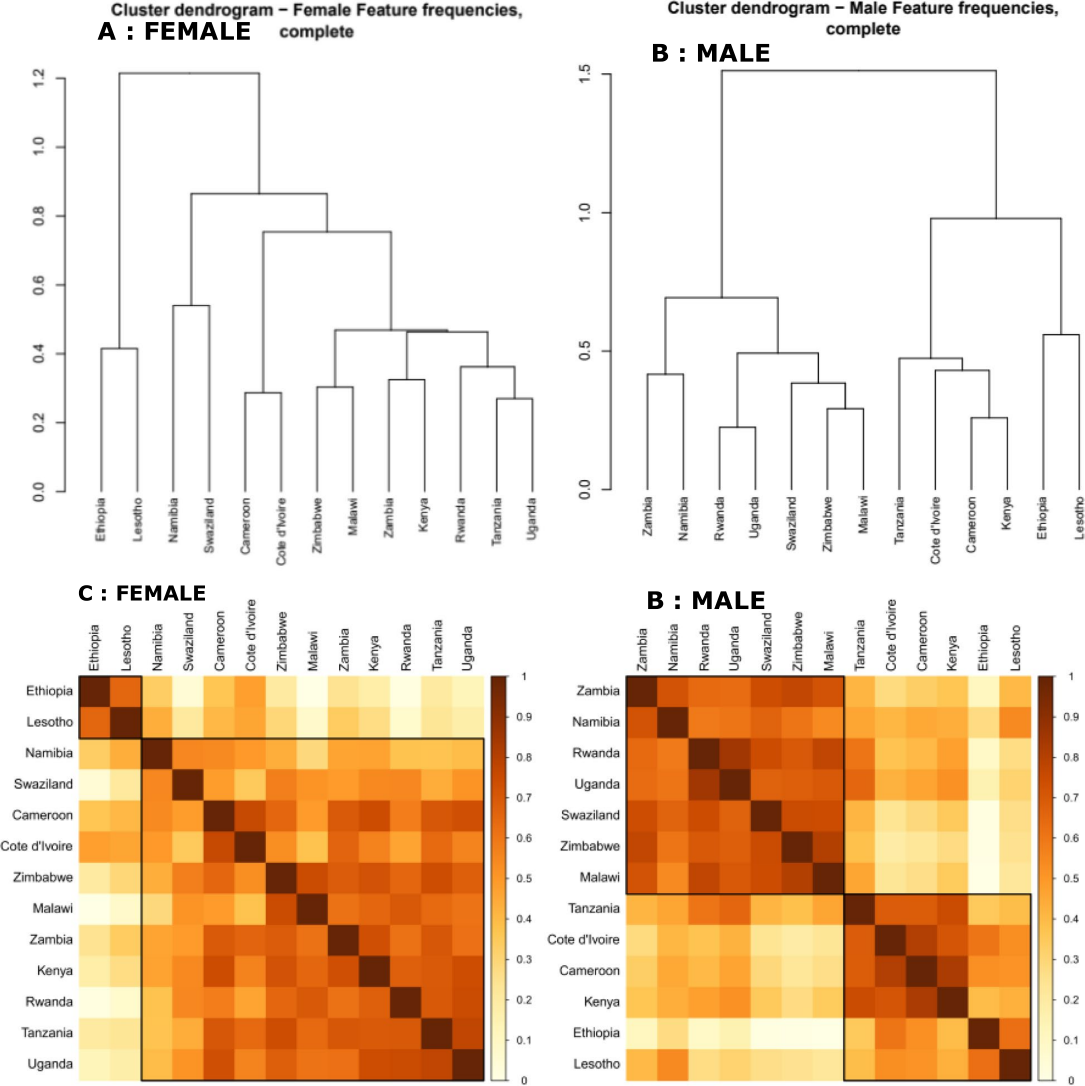
Cluster dendrograms (**A**: Females and **B**: Males) and Dissimilarity Matrix (**C**: Females, **D**: Males)

**Fig. 5:**
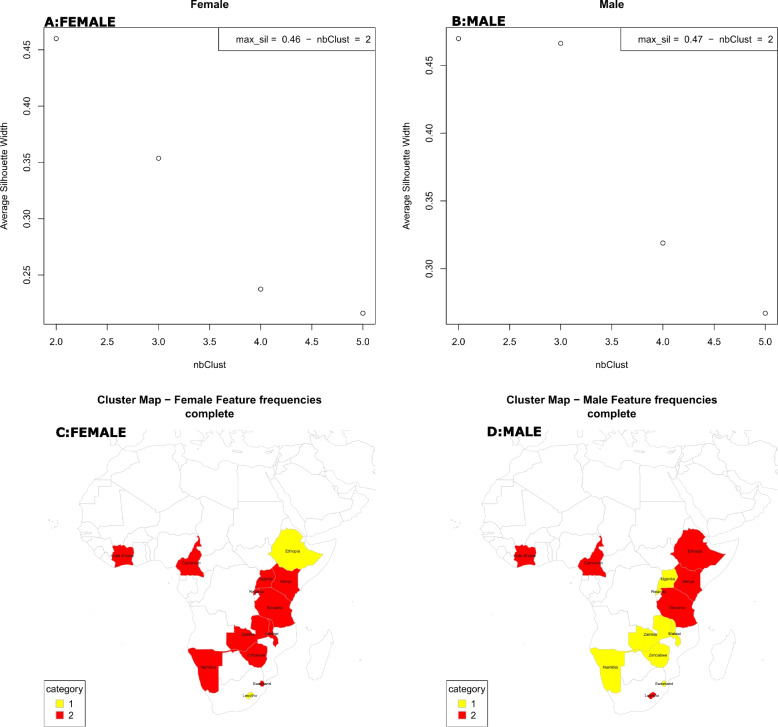
Highest optimal Silhouette Index for clusters and map of the selected clusters with countries (Red indicates cluster 1, yellow indicates cluster 2)

A dendrogram depicting the group of countries exhibiting similar features were generated employing hierarchical clustering to identify groups of countries with similar socio-behavioural characteristics (Fig. 4, A: Females, B: Males). The estimated Euclidean distance was also used to determine the pairwise countries' dissimilarity, yielding the Euclidean dissimilarity matrix**.** (Fig. 4, C: Females and D: Males). The silhouette index assesses the degree of isolation from other clusters of each data point within a group. Cluster misclassification indicates a -1 score, well-separated clusters have a 1 score, and 0 values indicate overlapped or ambiguous clusters. The maximum silhouette index was determined separately for males and females, providing optimal separation and compactness, which improves the clustering's quality. Consequently, the male and female scores are 0.46 and 0.47, respectively, (Fig. 5, A: Females and B: Males) generating two distinct categories of countries in clusters 1 and 2 ( Fig. 5, C: Females and D: Males).

**Fig. 6:**
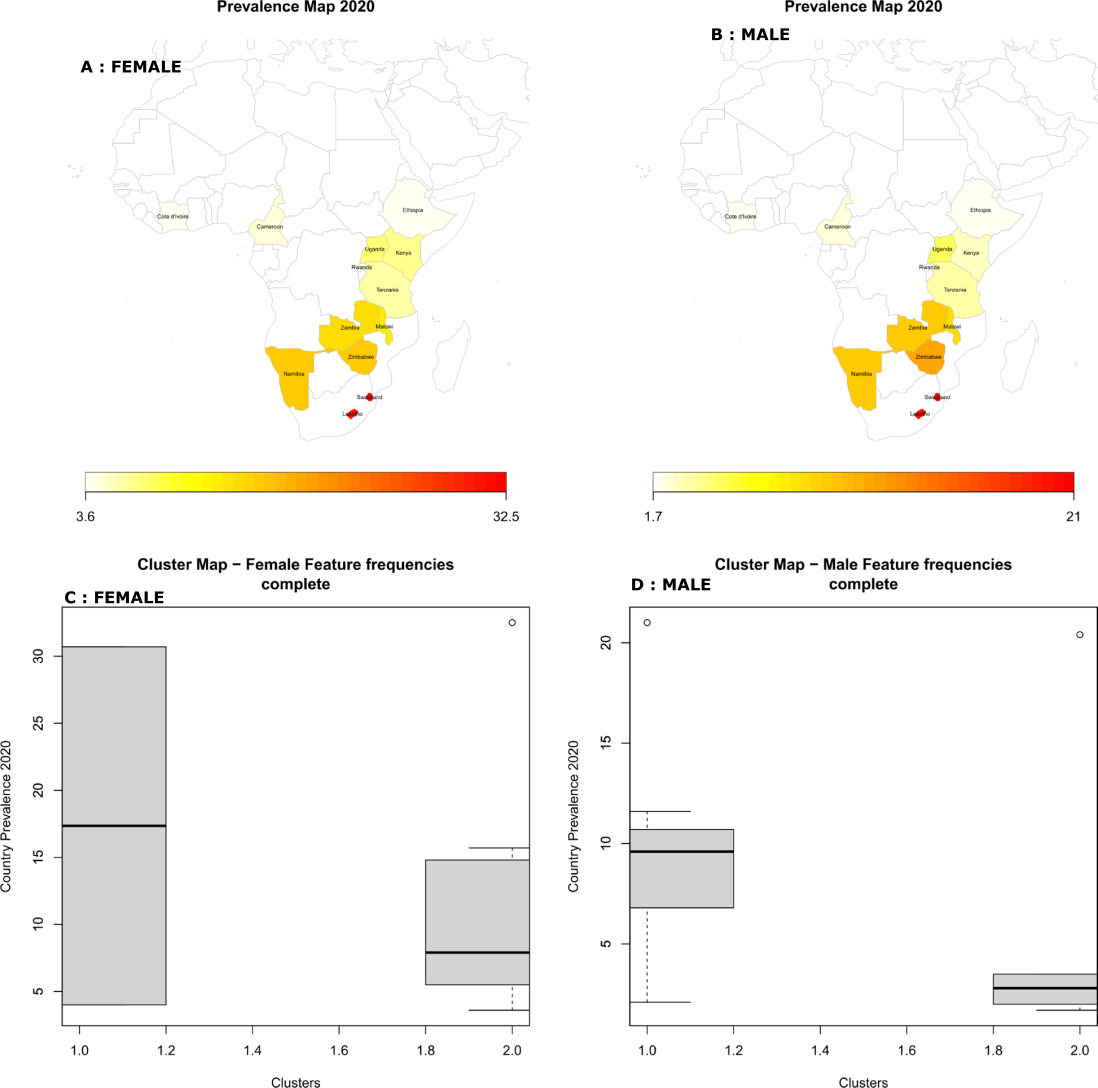
HIV prevalence distribution map per country (**A**: Female and **B**: Male) and cluster Boxplots for HIV positivity (Median) per cluster (**C**: Female and **D**: Male)

In the population of females, cluster 1 comprises Ethiopia and Lesotho (Fig. 5, C), yellow in colour, which have the highest median and average HIV prevalences of 17.35% and 17.35%, respectively (Fig. 6, A, and C). In this region, significant factors contributing to high prevalence levels are; 73.9% of females in this region were not enrolled in school, 80.2% are unaware of their HIV status, 70% are married or lived together, and less than half (42.0%) of them are the head of the family. 64.7% and 96.3% of them have had jobs in the last 12 months and are living in urban areas, respectively (Table S5, Figure S1, A).

Females in Cluster 2 include countries like Cameroon, Côte d'Ivoire, Kenya, Malawi, Namibia, Zambia, Rwanda, Swaziland, Tanzania, Uganda, and Zimbabwe (Fig. 5, C), red in colour. In this cluster, the average HIV prevalence is 11.16% and the median is 7.9% (Figs. 6, A, and C). In this cluster, 66.9% of the females live in rural areas, 72.2% of them are married or cohabiting,

67.8% have not held a paid job in the last 12 months, 77.5% are not in school, 94.0% are unaware of their HIV status, and 39.3% are the wives or partners of the household head. All these variables are significantly associated with HIV positivity (Table S5, Figure S1, C).

Cluster 1 countries for males include Zimbabwe, Namibia, Malawi, Rwanda, Uganda, Swaziland, Zambia, and (Fig. 5, D), yellow in colour. This cluster's male HIV prevalence is 9.67% (median 9.60%), (Figs. 6, B, and D). In this region, 53.9.0% of men are married, 67.5% are circumcised, delayed causing pregnancy (50.4%) and 66.9% live in rural areas. Furthermore, 50.1% had been jobless within the past 12 months and 48.6% are the head of the family, and they are all significantly contributing to HIV status (Table S5, Figure S1, B).

Countries like Cameroon, Cote d'Ivoire, Ethiopia, Kenya, Tanzania, and Lesotho are among those with males in cluster 2, (Figs. 5, B, and D). With an average HIV prevalence of 5.53% (2.8% median) among males, this cluster has the lowest rates of the disease (Figs. 6, B, and D). The region has a low HIV prevalence due to significant contribution of characteristics, including a high rate of male circumcision (86.1%), a sizable employment rate (57.2%), a population that is primarily urban (63.8%), married (57.5%) and a sizable percentage of homes led males (53.2%) (Table S5, Figure S1, D).

## Discussion

The countries grouped based on how comparable their identified socio-behavioural HIV predictors were using a dataset of over 300,000 respondents in 13 SSA countries.

To highlight the socio-behavioural commonalities among SSA countries and pinpoint the primary axes along which data variation is greatest, principal component analysis was employed to reduce the data's dimensionality from 20 and 26 to only two per sex. Then hierarchical clustering was used to identify groups of countries with similar socio-behavioural features. The method enabled us to isolate the first principal component, which explained 62.3% of the variation in socio-behavioural patterns in females and 70.1% in males across 13 countries, and the second principal component, which explained 18.3% and 20.6% of the variation in socio-behavioural patterns in females and males respectively, across 13 countries. It was also used to identify the most significant factors that affected the principal components for both sexes. For the first principal component, the most significant contributors were urban and rural residence, as well as the male circumcision status, while the second principal component's contributors included marital status, known HIV status, postponing or avoiding pregnancy, ever visiting a TB clinic for treatment, and family ties to the household head.

Using HIV prevalence maps and boxplots, the distribution trajectory between HIV predictors and clusters of HIV prevalence was discovered, illuminating an intuitive grasp of the relationships. Ethiopia and Lesotho are in cluster 1 and have a substantially greater HIV prevalence among females (17.35%) than the other cluster. In this region, 73.9% of females were not enrolled in school, 80.2% were not aware of their HIV status, 70% were married or cohabited, and fewer than half (42.0%) were the family's head of household. In the past 12 months, 64.7% of them had jobs, and 96.3% of them resided in metropolitan regions. Females individuals from Ethiopia and Lesotho have the biggest variation in HIV prevalence, at 26.7%, despite sharing many socio-behavioural features. Differences in cultural practices, social norms, and attitudes towards HIV prevention and treatment, as well as variations in healthcare resources and the implementation of prevention interventions, maybe a contribute to variations in HIV prevalence rates between females in Ethiopia and Lesotho, despite having similar socio-behavioural characteristics.

Most females are HIV-unaware and ignorant of HIV risk reduction strategies, which may be the explanation for this highest HIV prevalence in cluster 1. Living in urban areas and having more financial resources, may lead to more sexual partners. Most people living in urban areas appeared to be more at risk for the disease than those living in rural areas confirming the findings from Baranczuk et al. and Sing et al. studies [30, 31].

Females, individuals have an 11.16% HIV-positive rate in Cameroon, Côte d'Ivoire, Kenya, Malawi, Namibia, Zambia, Rwanda, Swaziland, Tanzania, Uganda and Zimbabwe. Here, 94.0% of the women are not aware of their HIV status, 77.5% are not enrolled in school, 72.2% of the women are married or living with someone else and 67.8% have not had paid employment in the last 12 months. The less educated women in this region are more dependent on their husbands for financial assistance because they have fewer formal work prospects and may have dropped out of school before getting married. More females are HIV-unaware here, which may be due to a lack of awareness of HIV risk reduction strategies and higher HIV risk asserting the findings in [32, 33]. There is a higher need for expanded HIV screening in these countries, which are mostly in eastern and southern Africa.

More than half (67.5%) of men in Namibia, Malawi, Rwanda, Uganda, Swaziland, Zambia and Zimbabwe are uncircumcised, which raises the risk of HIV infection and the spread of the disease and is consistent with other studies [34]. These might have contributed to the clusters’ 9.67% HIV positivity rate for men. More than half (66.9%) of them residing in rural areas may be contributing to lack of access to HIV testing and other prevention services, are unable to receive health care because 50% of them lack jobs, and are thus forced to engage in risky behaviour to make ends meet. Almost half (46.0%) of them are not married, making them vulnerable to promiscuity, reckless behaviour, and casual partnerships.

In contrast to the other cluster, Cameroon, Cote d'Ivoire, Ethiopia, Kenya, Tanzania and Lesotho are host to males with the lowest rates of HIV positivity (5.53%). The greatest rate of male circumcision (86.1%), a greater percentage of employment (57.2%), and the fact that more than half of them (57.5%) are married and are household heads (53.2%), could be linked to this low level of HIV which is consistent with studies [30].

While Merzouki et al.'s methodology was heavily borrowed in this study, which categorized countries according to general socio-behavioural traits from Demographic and Health Survey (DHS) data [26], we, however, used socio-behavioural predictors of HIV for screening that had already been established in the same region [27], as opposed to general indicators. In contrast to their work, which explained 69% of the variation but was constrained by the use of model estimates of HIV incidence that may deviate from reality, we analyzed used HIV prevalence estimates derived from the same data, reflecting the real relationship between the countries and the HIV prevalence estimates. We explained 62.3% and 70.1% total variation in the characteristics in females and males respectively.

One limitation of this study is its entire dependency on the generated data from the previous study, which suffered from a high degree of missingness and inconclusiveness from self-reported data that potentially impacted the training data [27]. To account for the significant variation in HIV prevalence between Ethiopia and Lesotho, a thorough analysis encompassing multiple factors is warranted. This analysis should thoroughly consider various aspects related to both countries, such as cultural, socioeconomic, and healthcare factors, in order to gain a comprehensive understanding of the underlying causes. To evaluate and compare the performance of the clustering approach used in this study, alternative clustering methods can be employed. This allows for a comprehensive assessment of different approaches and their effectiveness in clustering the data.

The study demonstrated that population-based surveys and clustering analysis guided by HIV predictors for screening might supply pertinent insights into the populations for HIV testing in SSA countries. This was a clear indication of general dissimilarity between countries. Sociobehavioural heterogeneity explained the spatial variation of the HIV epidemic at the regional level by comparing and analysing the SSA countries which would help in designing efficient treatments. Based on the socio-behavioural characteristics of the population, we split the region into groups that can inform actions and policies aimed at the general population as well as monitor the underlying causes of HIV infection. Finding groups of countries with comparable socio-behavioural traits can help formulate policy, share knowledge, encourage focused resource allocation, provide predictive analysis, promote cultural understanding, and improve diplomacy. These revelations can lead to better international cooperation, more effective development initiatives, and better outcomes for all societies.

## Supplementary Information

**Additional file 1.**

## Data Availability

The datasets used and/or analysed during the current study are available from this link https://phia-data.icap.columbia.edu/ on request.

## Abbreviations

SSA: sub-Saharan Africa
UNAIDS: The Joint United Nations Programme
HIV: Human Immunodeficiency Virus
AIDS: Acquired Immune Deficiency Syndrome
STI: Sexually Transmitted Infection
TB: Tuberculosis
PHIA: Population-based HIV Impact Assessment
DHS: Demographic and Health Surveys
PCA: Principal Component Analysis
ART: Antiretroviral therapy
