# Supplementary material for: Use of unsupervised machine learning to characterise HIV predictors in sub-Saharan Africa
Source: BMC Infect Dis. 2023 Jul 19;23:482. doi: 10.1186/s12879-023-08467-7 (PMC10354889; doi:10.1186/s12879-023-08467-7)
Supplement: Supplementary file 1 — Additional file 1. [file 12879_2023_8467_MOESM1_ESM.pdf]

Table S1: Country socio-behavioural variables for females

Country	Tanzania	Rwanda	Uganda	Cameroon	Zimbabwe	Zambia	Ethiopia	Malawi	Cote d'Ivoire	Namibia	Lesotho	Swaziland
Average.age.of.respondent	34.1	32.5	30.1	32.1	34.8	29.8	30.7	31	31.4	32.8	31.8	33.7
Not.Enrolled.in.school	81.1	76	78.1	73.3	83.7	78.7	70.7	78.7	69.9	72.3	77.2	74.9
Enrolled.in.school	8.4	18	16.5	19.7	13.1	19.1	22.6	16	16.5	25.9	22.5	22.1
not.work.for.payment.last.12.months	68.9	69.7	55.3	56.5	73.2	78.1	64.2	81.3	65.4	62.2	65.2	64.1
work.for.payment.last.12.months	31.1	30.3	44.7	43.5	26.8	21.9	35.8	18.7	34.6	37.8	34.8	35.9
Never.married.or.lived.together	23.1	35.6	25.7	29.5	22	30.8	29.4	21.7	33	57.8	30.5	47.5
Ever.married.or.lived.together	76.9	64.4	74.3	70.5	78	69.1	70.5	78.3	67	42.2	69.5	52.4
Average.no..of.times.been.pregnant	3.6	3	3.5	3.5	3.1	3.2	2.6	3.4	3.3	2.4	2.1	2.6
Average.number.of.children.given.birth.since.2012	0.7	0.7	0.8	0.7	0.6	0.8	0.7	0.7	0.6	18.3	0.5	0.5
Delaying.or.avoiding.getting.pregnant	28.9	35.6	34.5	43	45.9	52.3	29.3	49.8	29.8	51.3	51.9	54.4
Average.age.at.first.sex	17.5	19.5	17.1	17.2	18.6	17.5	18.1	17.4	17.3	18.3	18.2	18.2
Ever.visited.TB.clinic.for.treatment	3.5	6.2	4.8	4.3	9.6	5.7	5.8	5.1	2.8	14.5	20	16.9
Place.of.residence.rural	62.4	80.6	70.1	48.9	64.3	53.8	0	81	36.5	42.2	7.5	72.2
Place.of.residence.urban	37.6	19.4	29.9	51.1	35.7	46.2	100	19	63.5	57.8	92.5	27.8
Not.Known.HIV.Status	85.6	87.5	87	85	81.2	82.3	88.2	84	84.1	82.3	72.2	67.3
Known.HIV.Status	3.9	2.7	5.5	2.7	12.1	10.3	3.3	9.6	2	13.4	25.8	29.9
Average.wealthscorecont	0.5	0.3	0.5	0.5	0.5	0.5	0.5	0.3	0.3	0.6	0.5	0.4
Relationship.to.household.head.Brother.or.sister	2.4	5.3	5.5	5.6	3	2.7	3.7	1.6	5.9	5.6	3.5	3.1
Relationship.to.household.head.Grandchild	2.6	2.5	2.9	2	4.1	2.6	0.9	2.8	1.9	7.2	4.9	7.1
Relationship.to.household.head.Head	20.4	17.4	18.5	21	36.1	15.1	44.2	34.4	15.7	36.2	39.8	36.9
Relationship.to.household.head.Not.related	3.3	5.1	2.1	1.4	1.5	0.9	2.9	0.7	3.8	3.4	3.1	1.4
Relationship.to.household.head.Other.relative	5.2	7	8	8.5	5.6	7.7	3	3.1	3.8	10	4	6.1
Relationship.to.household.head.Parent	1.5	0.6	1.5	1.5	1.6	0.2	6.4	0.6	1.3	1	0.3	0.7
Relationship.to.household.head.Son.or.daughter	15.6	23.3	20.2	18.7	13.2	21.5	13.4	17.1	14.3	17.6	16.1	21.6
Relationship.to.household.head.Son.or.daughter-inlaw	3.6	1.2	3.4	3.9	4.6	1.3	0.3	0.3	2.7	1.5	3.6	4.8
Relationship.to.household.head.Wife.or.husband.or.partner	44.4	36.4	37	36.4	29.2	46.9	24.8	38.4	43.8	16.5	24.3	17

Table S2: Country socio-behavioural variables for males

Country	Tanzania	Rwanda	Uganda	Cameroon	Zimbabwe	Zambia	Ethiopia	Malawi	Cote d'Ivoire	Namibia	Lesotho	Swaziland	Kenya
Age.of.respondent	33.5	31.8	29.9	31.6	33.9	30.1	31.1	30.8	32.4	32	31.1	32.9	31.5
Did.not.work.for.payment.last.12.months	45.4	50.2	37.6	34.2	50	53.8	41.2	58.8	41.4	46.2	54.6	53.7	38.7
work.for.payment.last.12.months	54.6	49.8	62.4	65.8	50	46.2	58.8	41.2	58.6	53.8	45.4	46.3	61.3
Never.married.or.lived.together	35.9	45.3	40	45.7	38.4	44.3	41.5	35.7	46.2	60.5	47	58	42.2
Ever.married.or.lived.together	64.1	54.7	60	54.3	61.6	55.5	58.4	64.3	53.7	39.3	52.9	42	57.8
Delaying.or.avoiding.getting.pregnant	26.9	35.8	34.4	50.2	48.8	68.5	30.5	52.5	28.8	55.5	56.7	57.3	55.4
Circumcision.status.no	20.7	57.6	55.2	6.3	83.8	73	3.3	71.6	4.9	60.1	31.2	71.5	8.1
Circumcision.status	79.3	42.4	44.8	93.7	16.1	27	96.7	28.4	95.1	39.9	68.8	28.4	91.9
First.age.engaging.at.sex	17.8	19.7	17.7	18	19.4	17.6	19.8	17.6	18	17.8	17.8	18.7	17.3
Ever.visited.TB.clinic.for.treatment	4	7.4	5.2	5.1	10.4	7.5	6.9	4.1	3.6	14.7	19.8	13.1	9
Urban.area.indicator.rural	65.6	79.1	72.5	46.1	67	54.6	0	78.5	39.9	42.8	8.2	74.1	61.1
Urban.area.indicator.urban	34.4	20.9	27.5	53.9	33	45.4	100	21.5	60.1	57.2	91.8	25.9	38.9
wealthscorecont	0.5	0.3	0.4	0.5	0.5	0.5	0.4	0.3	0.3	0.6	0.5	0.4	0.4
Relationship.to.household.head.Brother.or.sister	3.2	5.1	5.6	7.8	4.4	4	4.1	3.3	7.5	6.2	3.7	3.5	2
Relationship.to.household.head.Grandchild	3.1	2.6	4.1	3	6.7	3.8	1	3.5	2	9.1	6	9.6	3.4
Relationship.to.household.head.Head	57	49.9	49.8	50.2	49.1	53.5	52.9	54.8	58.6	41.8	50.4	41.4	55.6
Relationship.to.household.head.Not.related	3.7	6.7	3.1	2.2	2.4	1	1.6	1.2	3.6	4.7	3.9	1.6	3.8
Relationship.to.household.head.Other.relative	6.7	5.6	5.9	9.6	6.1	9.3	2.6	4	2.6	11.6	4.8	6.8	4.1
Relationship.to.household.head.Son.or.daughter	24.1	25.6	26.9	24	21.6	26	19	21.9	17	18.7	25.5	28.5	27
Relationship.to.household.head.Wife.or.husband.or.partner	1	3.5	2.4	1.5	7.6	1.1	14.2	10.1	0.4	5.5	5	5.8	3.2

Table S3: Females Principal components loadings

	PC1	PC2
Age.of.respondent	0.002013	-0.00797
Did.not.work.for.payment.last.12.months	0.089226	0.131577
work.for.payment.last.12.months	-0.08923	-0.13158
Never.married.or.lived.together	0.006809	0.110866
Ever.married.or.lived.together	-0.00654	-0.11279
Delaying.or.avoiding.getting.pregnant	0.102701	0.233244
Circumcision.status.no	0.569804	0.343184
Circumcision.status	-0.57017	-0.3434
First.age.engaging.at.sex	0.00034	0.005602
Ever.visited.TB.clinic.for.treatment	0.004972	0.127601
Urban.area.indicator.rural	0.40002	-0.55642
Urban.area.indicator.urban	-0.40002	0.556416
wealthscorecont	-7.00E-05	0.001544
Relationship.to.household.head.Brother.or.sister	-0.00722	-0.0032
Relationship.to.household.head.Grandchild	0.026763	0.042236
Relationship.to.household.head.Head	-0.04256	-0.08398
Relationship.to.household.head.Not.related	-0.00242	-0.01349
Relationship.to.household.head.Other.relative	0.01818	0.018497
Relationship.to.household.head.Son.or.daughter	0.026162	-0.02663
Relationship.to.household.head.Wife.or.husband.or.partner	-0.00207	0.06783

Table S4: Females Principal Components loadings

	PC1	PC2
Average.age.of.respondent	-0.00809	-0.02538
Not.Enrolled.in.school	-0.05825	0.029709
Enrolled.in.school	0.064238	-0.14672
not.work.for.payment.last.12.months	-0.06058	0.046618
work.for.payment.last.12.months	0.060581	-0.04662
Never.married.or.lived.together	0.047095	-0.41878
Ever.married.or.lived.together	-0.04739	0.419222
Average.no..of.times.been.pregnant	-0.00773	0.015572
Average.number.of.children.given.birth.since.2012	0.022041	-0.13759
Delaying.or.avoiding.getting.pregnant	-0.0435	-0.33928
Average.age.at.first.sex	-0.0003	-0.01346
Ever.visited.TB.clinic.for.treatment	0.047839	-0.22374
Place.of.residence.rural	-0.68528	-0.10955
Place.of.residence.urban	0.685284	0.109553
Not.Known.HIV.Status	-0.02788	0.217801
Known.HIV.Status	0.034923	-0.34541
Average.wealthscorecont	0.001124	-0.00094
Relationship.to.household.head.Brother.or.sister	0.007937	-0.00251
Relationship.to.household.head.Grandchild	-0.00203	-0.08946
Relationship.to.household.head.Head	0.138054	-0.25603
Relationship.to.household.head.Not.related	0.008596	0.005086
Relationship.to.household.head.Other.relative	-0.01402	-0.04162
Relationship.to.household.head.Parent	0.024404	0.02399
Relationship.to.household.head.Son.or.daughter	-0.048	-0.05368
Relationship.to.household.head.Son.or.daughter.inlaw	-0.00537	-0.01441
Relationship.to.household.head.Wife.or.husband.or.partner	-0.11106	0.408927

Table S5: Significant test of variables contribution to HIV positivity in each cluster 1 per sex

**Cluster 1 for females**					
	**2.50%**	**97.50%**	**Odds Ratio**	**p values**	**significant?**
Enrolled in school	0.042893	0.05459	0.048389	0.000	significant
work for payment last 12 months	0.261437	0.297883	0.279066	0.000	significant
Ever married or lived together	0.063794	0.070062	0.066855	0.000	significant
Ever visited TB clinic for treatment	0.778255	0.997379	0.881031	0.045	significant
Urban area indicator	0.338958	0.376065	0.35703	0.000	significant
Known HIV Status	12905.6089	19789.79987	15981.22077	0.000	significant
Relationship to household head_Head	0.58271	0.675562	0.627421	0.000	significant
**Cluster 2 for females**					
	**2.50%**	**97.50%**	**Odds Ratio**	**p values**	**significant?**
Ever married or lived together	0.091877	0.102328	0.096962	0.000	significant
Delaying or avoiding getting pregnant	0.278069	0.30865	0.292961	0.000	significant
Urban area indicator	0.191116	0.210283	0.20047	0.000	significant
Known HIV Status	17787.0513	26930.42598	21886.36261	0.000	significant
Relationship to household head_Wife/husband/partner	0.463206	0.547072	0.503396	0.000	significant
**Cluster 1 for males**					
	**2.50%**	**97.50%**	**Odds Ratio**	**p values**	**significant?**
work for payment last 12 months	0.357967	0.384097	0.370802	0.000	significant
Ever married or lived together	0.505589	0.537726	0.52141	0.000	significant
Delaying or avoiding getting pregnant	0.540686	0.570438	0.555363	0.000	significant
Circumcision status	0.118674	0.128165	0.123328	0.000	significant
Urban area indicator	0.793584	0.841821	0.817347	0.000	significant
**Cluster 2 for males**					
**2.50%**	**97.50%**	**Odds Ratio**	**p values**	**significant?**	
work for payment last 12 months	0.206297	0.219407	0.212751	0.000	significant
Circumcision status	0.093566	0.100666	0.097051	0.000	significant
Urban area indicator	0.628562	0.665777	0.646902	0.000	significant

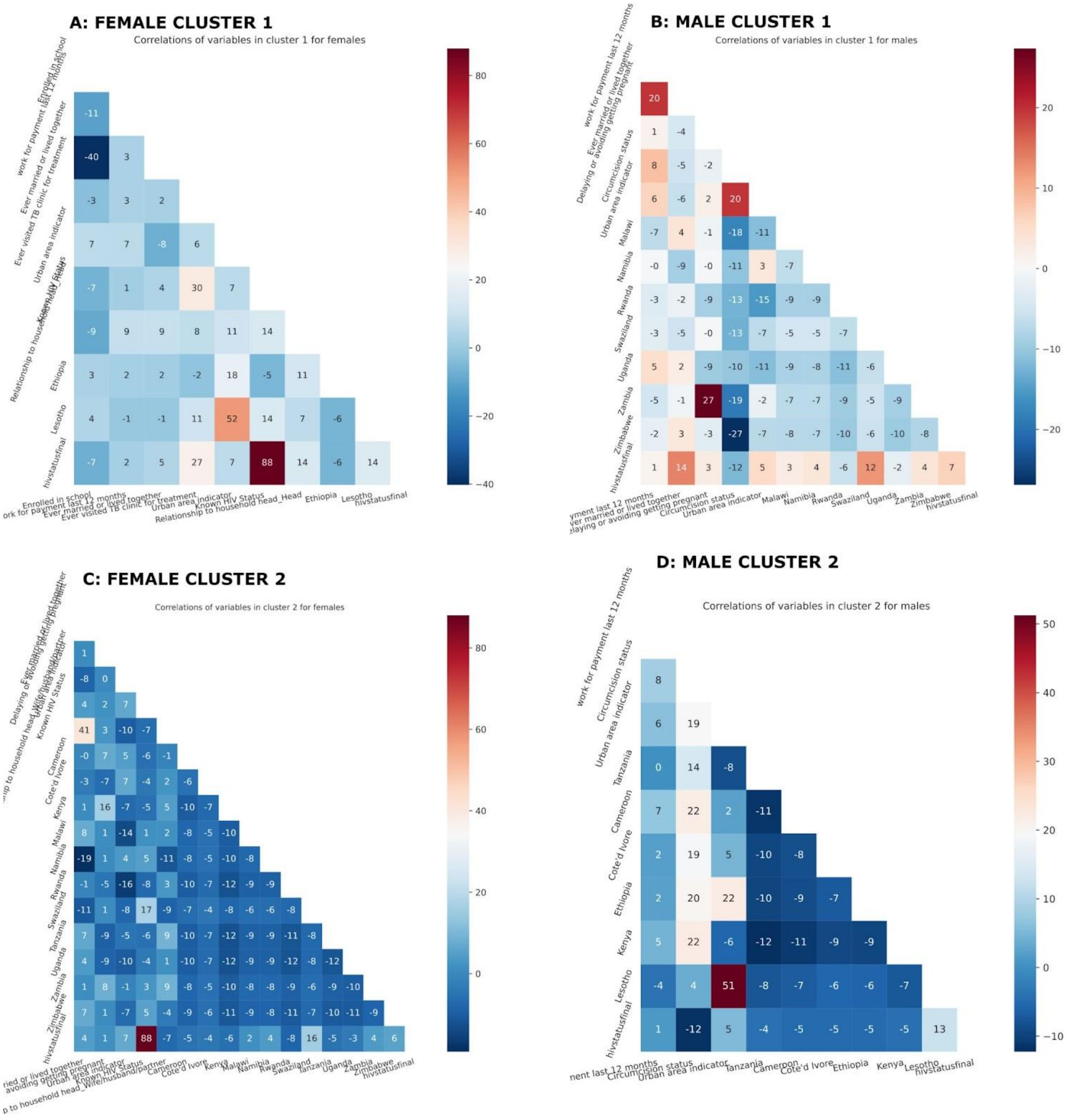

Figure S1: Heatmap displaying the correlation between variables and HIV positivity sex per cluster
